# Artificial Intelligence‐Based Analysis of Coronary Atherosclerotic Plaque on Intravascular Ultrasound: A Systematic Review and Meta‐Analysis

**DOI:** 10.1002/clc.70423

**Published:** 2026-07-21

**Authors:** Pooya Eini, Homa Serpoush, Mohammad Rezayee, Milan Kassulke

**Affiliations:** ^1^ Cardiovascular Imaging Research Center Rajaie Cardiovascular Institute Tehran Iran; ^2^ Hamadan University of Medical Sciences Hamadan Iran; ^3^ College of Human Medicine Michigan State University East Lansing Michigan USA

**Keywords:** artificial intelligence, coronary atherosclerosis, intravascular ultrasound, machine learning, plaque detection

## Abstract

**Background:**

Coronary atherosclerotic plaque detection is essential for the diagnosis and management of coronary artery disease. Although intravascular ultrasound (IVUS) enables detailed plaque assessment, its clinical use is limited by time‐consuming interpretation and observer variability. Artificial intelligence (AI)‐based methods offer a promising approach to automate IVUS plaque detection. This systematic review and meta‐analysis evaluated the diagnostic performance of AI models for IVUS‐based coronary plaque detection.

**Methods:**

A comprehensive literature search was conducted across major databases from inception to December 2025. Data extraction and risk of bias assessment were performed independently using the PROBAST‐AI tool. Pooled sensitivity and specificity were estimated using bivariate random‐effects models, and sources of heterogeneity were explored through meta‐regression. Certainty of evidence was assessed using the GRADE framework.

**Results:**

Among 10 included studies (six for meta‐analysis), the pooled sensitivity and specificity were both 0.99 (95% CI, 0.93–1.00 and 0.96–1.00, respectively), with an area under the summary receiver operating characteristic curve of 1.00 (95% CI, 0.99–1.00). Moderate between‐study heterogeneity was observed (generalized *I* ^2^ = 46.1%), affecting both sensitivity and specificity. Meta‐regression analyses identified sample size as a significant contributor to heterogeneity. Risk of bias assessment revealed a moderate overall risk, and the certainty of evidence was rated as moderate.

**Conclusions:**

AI‐based IVUS analysis shows promising performance for automated plaque and calcification assessment in research settings. However, limitations related to study design, sample size, and reporting transparency remain. Future research should prioritize large, multicenter, prospective studies and strict adherence to TRIPOD + AI guidelines to support clinical translation.

## Summary

- AI‐based analysis of IVUS images demonstrates excellent diagnostic accuracy for coronary plaque detection, with high sensitivity and specificity.

## Graphical abstract

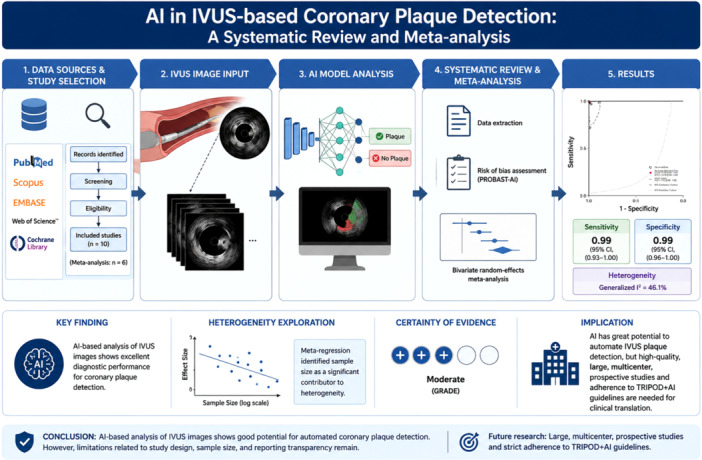

## 1 Introduction

Cardiovascular diseases (CVDs) remain the predominant cause of mortality globally, accounting for an estimated 19.2 million deaths in 2023 and representing approximately 32% of all deaths worldwide [1]. Coronary artery disease (CAD), a major subset of CVDs, is primarily driven by atherosclerosis, a chronic inflammatory process characterized by the accumulation of lipids, fibrous elements, and calcified plaques within the arterial intima‐media layer [2]. This plaque buildup leads to vessel lumen narrowing, reduced blood flow, and heightened risk of rupture, thrombosis, and acute cardiovascular events [3].

Coronary artery calcification, a marker of advanced atherosclerosis, is highly prevalent in patients with CAD, with angiographic evidence in 25%–30% of those undergoing percutaneous coronary intervention (PCI) [4]. Its presence not only complicates interventional procedures but also serves as a prognostic indicator for adverse outcomes [5]. Early and accurate detection of calcified plaques is thus essential for risk stratification, treatment planning, and improving patient prognosis.

Intravascular ultrasound (IVUS) is an established intracoronary imaging modality that provides cross‐sectional assessment of vessel architecture, plaque burden, and calcification, and is widely used to guide complex PCI [6]. IVUS enables the identification of calcified plaques as hyperechoic regions with acoustic shadowing, facilitating precise assessment of luminal stenosis and guiding PCI [7]. However, manual analysis of IVUS pullbacks often comprising hundreds of frames per patient, is labor‐intensive, time‐consuming, and subject to inter‐ and intra‐observer variability, limiting its efficiency in clinical practice [8].

The integration of artificial intelligence (AI), particularly deep learning algorithms such as convolutional neural networks (CNNs), ResNet, U‐Net, and ensemble models, has shown considerable promise in automating ultrasound image interpretation [9, 10]. These models leverage transfer learning, feature extraction, and segmentation techniques to detect plaque components, including calcification, with high accuracy [2]. Preliminary studies report good performance metrics, but results vary widely due to differences in data sets, model architectures, and validation methods.

Despite the growing body of evidence, no comprehensive synthesis has evaluated the overall performance of AI models in coronary artery plaque detection using IVUS. Heterogeneity in study designs, patient populations, and reporting standards hinders direct comparisons and clinical translation. This systematic review and meta‐analysis aims to address this gap by systematically appraising the literature and quantitatively pooling diagnostic performance metrics across eligible studies. By identifying sources of variability and assessing model generalizability, we seek to provide evidence‐based insights into the efficacy of AI in IVUS‐based plaque detection, informing future research directions and facilitating its integration into routine cardiovascular care.

## 2 Methods

This systematic review and meta‐analysis were conducted and reported in accordance with the Preferred Reporting Items for Systematic Reviews and Meta‐Analyses of Diagnostic Test Accuracy Studies (PRISMA) [11]. Study protocol registered in PROSPERO (ID: CRD420251267734).

### 2.1 Search Strategy

A comprehensive systematic literature search was performed across multiple electronic databases, including PubMed/MEDLINE, Embase, Scopus, Web of Science, and Embase, from database inception until December 1, 2025. The search strategy was developed around three core concepts:

1. AI methodologies (machine learning, deep learning, CNNs).
2. IVUS imaging.
3. Coronary artery plaque detection (with emphasis on calcification or atherosclerotic plaque).

Controlled vocabulary terms (MeSH in PubMed, Emtree in Embase) and free‐text keywords were combined using Boolean operators (AND/OR). No language restrictions were applied. Supplementary searches included screening of reference lists from included studies and relevant reviews. The full search strategies for each database are provided in Supporting Information S1: Table 1 for reproducibility.

### 2.2 Eligibility Criteria

Inclusion and exclusion criteria were predefined using the PICOS framework (Population, Intervention, Comparator, Outcomes, Study design).

Population—Patients undergoing IVUS imaging for suspected or confirmed CAD, including those with atherosclerotic plaques or calcification; studies involving human‐derived IVUS images.

Intervention—AI‐based models for detection, classification, or segmentation of coronary artery plaques in IVUS images.

Comparator—Not mandatory; where reported, conventional manual annotation, non‐AI methods, or ground truth from expert radiologists/cardiologists.

Outcomes—Diagnostic performance metrics including accuracy, sensitivity (recall), specificity, precision, F1‐score, Dice coefficient, intersection over union (IoU), and area under the curve (AUC).

Study design—Original research studies reporting diagnostic test accuracy of AI models on IVUS images, including retrospective cohort studies, validation studies, and conference papers with sufficient methodological detail.

The target condition was coronary atherosclerotic plaque identified on IVUS, with particular emphasis on calcified plaque when reported. Because the included literature used heterogeneous task definitions, we grouped studies into three task categories: calcified plaque detection, plaque‐type classification, and plaque/calcification segmentation. The quantitative diagnostic meta‐analysis was restricted to studies that provided sufficient data to reconstruct 2 × 2 tables for a binary target condition, most commonly calcified versus non‐calcified plaque. Studies reporting only segmentation metrics, Dice coefficient, IoU, or non‐binary classification outcomes were summarized narratively.

Exclusion criteria comprised: non‐original research (reviews, editorials); animal or experimental (non‐human) studies; studies focused solely on other imaging modalities (optical coherence tomography [OCT] without IVUS co‐registration); and studies using non‐AI approaches exclusively.

### 2.3 Data Extraction

Two independent reviewers extracted data using a standardized, piloted form in Excel software. Discrepancies were resolved through discussion or adjudication by a third senior reviewer. Extracted items included: study characteristics (author, year, country, design); model details (algorithm type, architecture, transfer learning use, optimization); population characteristics (sample size, patient demographics where reported); data set details (number of images/patients, public/private); performance metrics (as listed above); and validation methods. Where studies reported multiple models or thresholds, the best‐performing model (highest primary metric, typically accuracy or AUC) or the author‐designated primary model was selected for meta‐analysis.

### 2.4 Risk of Bias and Certainty Assessment

Risk of bias was assessed independently by two reviewers using the Prediction Model Risk of Bias Assessment Tool adapted for AI (PROBAST‐AI), evaluating domains across participant selection, predictors, outcomes, and analysis. Disagreements were resolved by consensus or third‐reviewer input [12]. Overall risk of bias was classified as low, moderate, or high. The certainty of evidence was appraised using the Grading of Recommendations Assessment, Development and Evaluation (GRADE) framework for diagnostic tests, considering risk of bias, inconsistency, indirectness, imprecision, and publication bias.

### 2.5 Statistical Analysis

The primary index model from each study was selected as described. Analyses were performed in R (version 4.5) using the meta, metafor, and mada packages. A bivariate random‐effects model was employed to pool sensitivity and specificity, accounting for heterogeneity and the inherent correlation between these metrics. Hierarchical summary receiver operating characteristic (HSROC) curves were generated to summarize overall diagnostic performance. Heterogeneity was quantified using *I* ^2^ and tau^2^ statistics, with restricted maximum likelihood estimation (REML). Publication bias was assessed visually via Deeks’ funnel plot asymmetry and statistically using Deeks’ test. Sensitivity analyses involved leave‐one‐out diagnostics and exclusion of studies at high risk of bias. Meta‐regression was conducted to explore potential sources of between‐study heterogeneity by incorporating algorithm category (model type), validation strategy, sample size (log‐transformed), and event rate as prespecified moderators. Statistical significance was set at *p* < 0.05. Results were visualized using forest plots and HSROC curves.

## 3 Results

### 3.1 Study Selection

**Figure 1:**
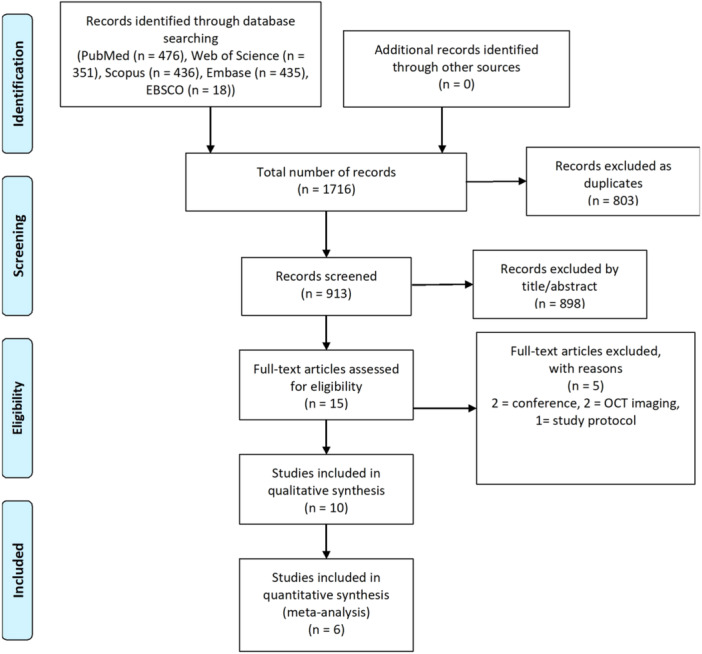
PRISMA flow diagram for study selection.

A comprehensive and systematic literature search identified a total of 1716 records across five electronic databases, including PubMed (*n* = 476), Web of Science (*n* = 351), Scopus (*n* = 436), Embase (*n* = 435), and EBSCO (*n* = 18). After removal of 803 duplicate records, 913 unique citations remained and were screened at the title and abstract level for relevance. This initial screening resulted in 15 articles being selected for full‐text assessment. Following detailed evaluation, five studies were excluded for predefined reasons: two were conference papers without full peer‐reviewed data, two employed OCT imaging rather than the prespecified imaging modality, and one was a study protocol lacking original outcome data. Consequently, 10 studies met all inclusion criteria and were included in the qualitative synthesis. Of these, six studies reported sufficient data on diagnostic performance and were therefore included in the quantitative meta‐analysis [13] (Figure 1).

### 3.2 Baseline Characteristics of Included Studies

All studies were retrospective in design and conducted across multiple geographic regions, including Asia (*n* = 7), Europe (*n* = 2), and multinational cohorts (*n* = 1), reflecting a broad international research effort.

Sample sizes varied substantially across studies. At the patient level, cohorts ranged from 10 to 80 patients, while image‐based data sets included between 100 and 8914 IVUS images or frames. Several studies relied on publicly available data sets, most notably Data set B from the MICCAI 2011 Challenge, which comprised 2175 images from 10 patients and was used in multiple investigations. Validation strategies were heterogeneous, including random train–test splits (*n* = 3), k‐fold cross‐validation (*n* = 4), leave‐one‐subject‐out cross‐validation (*n* = 1), and pullback‐based partitioning (*n* = 1).

Reporting of demographic characteristics was limited. Mean age was not reported in any of the included studies, and sex distribution was reported in only one study, which included 56% male participants. All studies enrolled patients with established CAD or atherosclerosis, including subsets undergoing PCI or presenting with stable angina. Event rates, defined as the proportion of images or frames containing calcification, varied widely across data sets, ranging from approximately 24% to 59%, with several studies intentionally enriched for calcified lesions (Table 1).

**Table 1:** Baseline characteristics.

First author/year	Type of study	Country	Validation	Selected features	Sample size	Male%	Past medical history	Event rate	Strengths	Weaknesses
Balocco et al. (2018) [14]	Calcified plaque detection in IVUS sequences using convolutional nets	Spain, Chile, Canada	Pullback‐based partition (36 training, 8 validation, 36 test pullbacks) with 10 train/test splits	Automatically learned features by convolutional neural networks	80 patients, 8914 IVUS images	—	Patients with atherosclerosis	40.65% of frames contained calcification	Preliminary sequence analysis	Low metrics; no large‐scale validation
Sofian et al. (2018) [15]	Calcification detection in IVUS images using deep feature learning	Malaysia	k‐fold cross‐validation (k = 2, 3, 5, 10)	Deep features from ResNet‐50, ResNet‐101, and Inception‐V3	Data set B from MICCAI Challenge 2011 (2175 IVUS images from 10 patients to 530 with calcification, 1645 without)	—	Patients with coronary artery disease	530 out of 2175 images (24.37%) contained calcification	High accuracy in polar format	Small scale; no artifacts considered
Li et al. (2021) [16]	Automatic detection of atherosclerotic plaque and calcification from IVUS images using deep CNNs	Taiwan	Leave‐one‐subject‐out cross‐validation (18 iterations)	Automatically learned features using three modified U‐Nets with cascaded networks	713 grayscale IVUS images from 18 patients	56% men, 33% women, 11% unknown	Patients with atherosclerosis/coronary artery disease	Approximately 78% of images contained calcification	Efficient for calcification; graph structure	Binary; limited multi‐class
Shinohara et al. (2021) [17]	Automatic detection of vessel structure by deep learning using IVUS images of coronary arteries	Japan	Random selection of test set (323 images from two patients) and training set (3415 images from 22 patients)	Automatically learned features using U‐Net	3738 IVUS images from 24 patients	—	Patients with stable angina pectoris	2209 out of 3738 images (59.1%) showed calcification, 459 (12.3%) showed a stent	Handles complex lesions/artifacts; multi‐component detection	Lower IoU for stents; single‐center
Sofian et al. (2021) [18]	Calcification detection for IVUS image using a direct acyclic graph architecture	Malaysia	k‐fold cross‐validation (k = 2, 3, 5, 10)	Deep features from DAGNet‐27 (27 layers of CNNs)	Data set B from MICCAI Challenge 2011 (2175 IVUS images from 10 patients to 530 with calcification, 1645 without)	—	Patients with coronary artery disease	530 out of 2175 images (24.37%) contained calcification	Accurate lumen detection (IoU 0.86)	Poor stent performance; retrospective bias
Archana and Vanithamani (2022) [19]	Segmentation of intima‐media thickness in IVUS images for detection of atherosclerosis	India	Not clearly specified (comparison with ground truth)	Total Variance Regularization, CLAHE, morphological operations	100 B‐mode IVUS images	—	Patients with atherosclerosis	—	Good for IMT measurement in stenosis	Limited data set; no segmentation of borders
Gao et al. (2022) [20]	IVUS image plaque recognition based on an improved Resnet network	China	Training set (480 images) and test set (240 images)	Automatically extracted features using deep learning	720 IVUS images (480 for training, 240 for testing)	—	Patients with atherosclerosis/coronary artery disease	240 test images with 80 images of each plaque type (calcified, fibrous, lipid)	High accuracy in multi‐class plaque recognition; efficient feature extraction	Limited data set; no segmentation of borders
Arora et al. (2023) [21]	CADNet: an advanced architecture for automatic detection of coronary artery calcification and shadow border in IVUS images	India	Random split (80% training, 20% testing)	Automatically learned features using CADNet (U‐Net with CBAM and ASPP modules)	1097 IVUS images from 12 patients (877 for training, 220 for testing)	—	Patients diagnosed with coronary artery disease	Data set focused on severely calcified lesions (Class III, IV with > 180° arc)	Near‐perfect classification; polar/Cartesian handling	Binary focus (calcified vs. non); small validation set
Arora et al. (2023) [13]	Calcification detection in IVUS images using a transfer learning‐based MultiSVM model	India	10‐fold cross‐validation	Deep features extracted by pre‐trained AlexNet	14 IVUS pullbacks from 10 patients (10 for training, 4 for testing)	—	Patients with coronary artery disease	Images classified as healthy, mild calcification (< 180° arc), or dense calcification (> 180° arc)	Superior in artifacts/shadows; real‐time augmentation	Single‐center; limited to 40 MHz IVUS
Prajapati et al. (2025) [8]	Automated diagnosis of atherosclerosis using multi‐layer ensemble models and bio‐inspired optimization in IVUS imaging	India	70:30 and 60:40 training and testing set proportions	Wiener filter‐based pre‐processing, Faster RCNN‐based segmentation, ShuffleNet‐v2 feature extraction	Data set B from MICCAI Challenge 2011 (2175 IVUS images from 10 patients to 530 with calcification, 1645 without)	—	Patients with atherosclerosis	530 out of 2175 images (24.37%) contained calcification	Robust hyperparameter tuning	Computationally intensive; no segmentation

CNNs formed the methodological foundation in most studies, including standard CNN architectures with varying depths, residual networks such as ResNet‐18, ResNet‐34, ResNet‐50, and ResNet‐101, and customized deep architectures designed specifically for IVUS data. Several studies employed U‐Net–based models and their variants, including attention U‐Net, UNet++, multi‐resolution U‐Net, and hybrid architectures incorporating convolutional block attention modules and atrous spatial pyramid pooling for segmentation tasks. In addition, one study introduced a directed acyclic graph‐based CNN architecture, while another used cascaded modified U‐Nets optimized with Dice, Tversky, and focal loss functions.

Traditional machine learning classifiers were frequently integrated with deep feature extractors, particularly in earlier studies. Support vector machines, k‐nearest neighbors, decision trees, naïve Bayes, and discriminant analysis classifiers were applied to features extracted from pretrained networks such as AlexNet, Inception‐V3, and ResNet. Transfer learning was commonly used to address limited data set sizes, with pretrained models fine‐tuned on IVUS images. More recent studies explored ensemble and hybrid frameworks, combining region‐based convolutional networks for localization, lightweight architectures for feature extraction, and bio‐inspired optimization techniques for classification. Performance metrics of included models are listed in Table 2.

**Table 2:** Performance metrics of artificial intelligence models for IVUS‐based coronary plaque analysis.

Author (year)	AI model	Accuracy (%)	Sensitivity/recall (%)	Specificity (%)	Precision (%)	F1‐Score (%)	Other metrics
Balocco et al. (2018) [14]	Convolutional neural networks (various depths)	—	83 (recall)	—	77	67	—
Sofian et al. (2018) [15]	ResNet‐50/101, Inception‐V3 + classifiers (SVM)	Up to 100	Up to 100	Up to 100	Up to 100	—	Excellent AUC in ROC
Sofian et al. (2021) [18]	27‐layer CNN with Directed Acyclic Graph (DAG)	98.16 (Cartesian), 99.08 (polar)	—	—	—	—	—
Li et al. (2021) [16]	Cascaded modified U‐Nets (Dice/Tversky/Focal loss)	> 90 (for borders/lumen)	> 90 (for borders/lumen)	> 90 (for borders/lumen)	> 90 (for borders/lumen)	—	AP 0.73 (calcification, focal loss)
Shinohara et al. (2021) [17]	U‐Net	97−98 (for clinically significant cases)	—	—	—	—	Mean IoU 0.66; Dice 0.73; Lumen correlation *ρ* = 0.97
Archana and Vanithamani (2022) [19]	Multi‐level set‐based segmentation (compared to Otsu, Active Contour, Watershed)	—	—	—	—	—	Highest JAC, Dice, Kappa, RI; lowest VOI, GCE with Multi‐Level Set
Gao et al. (2022) [20]	Improved ResNet (SE+ResNet18)	87.1	—	—	—	—	Superior to SVM (75.3%)
Arora et al. (2023) [13]	Transfer learning improved AlexNet + MultiSVM	99.8	—	—	—	99.64	GMean 99.80
Arora et al. (2023) [21]	CADNet (U‐Net with CBAM+ASPP)	—	87.74	—	87.68	—	mIoU 0.7894; Dice 0.8763
Prajapati et al. (2024) [8]	AAPC‐HALODL (Faster R‐CNN + ShuffleNet‐v2 + HALO optimization + SAE‐DELM ensemble)	98.33	98.33	—	97.87	98.1	—

### 3.3 Pooled Performance Metrics

**Figure 2:**
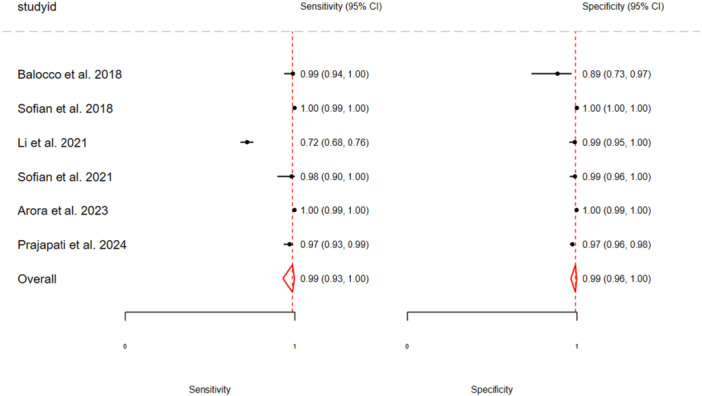
Forest plot for sensitivity and specificity.

**Figure 3:**
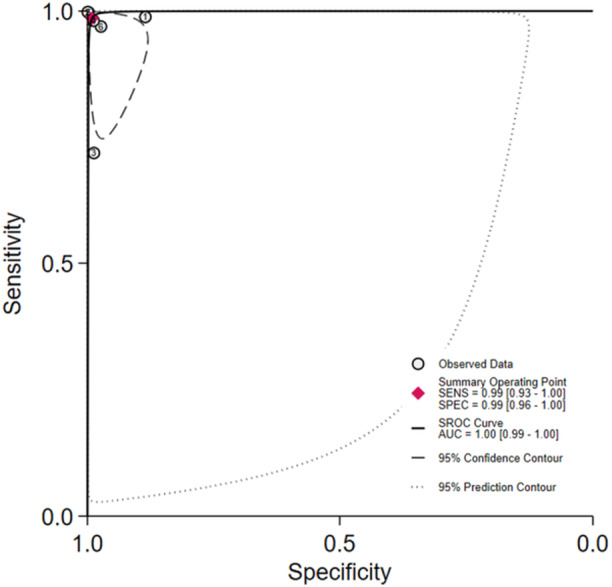
Receiver operating characteristic curve.

The quantitative synthesis included six studies that reported sufficient data to construct 2 × 2 contingency tables. Using a random‐effects model, the pooled sensitivity was 0.99 (95% confidence interval [CI], 0.93–1.00) and the pooled specificity was 0.99 (95% CI, 0.96–1.00) (Figure 2). The summary receiver operating characteristic (SROC) analysis yielded an area under the ROC curve (AUROC) of 1.00 (95% CI, 0.99–1.00), indicating a high level of overall diagnostic discrimination (Figure 3). Between‐study heterogeneity was moderate. The generalized *I* ^2^ was 46.12%, with *I* ^2^ values of 55.39% for sensitivity and 45.35% for specificity, suggesting variability across studies. Partial data set overlap was present because several studies used the MICCAI 2011 Data set B, reducing study independence and potentially inflating pooled accuracy. Therefore, the pooled estimates should be interpreted as reported performance under selected research conditions rather than definitive evidence of independent real‐world diagnostic accuracy.

### 3.4 Leave‐One‐Out Analysis

A leave‐one‐out sensitivity analysis was performed to evaluate the influence of individual studies on the pooled effect. Sequential omission of each study did not materially alter the overall pooled estimate, with effect sizes remaining statistically significant across all iterations. The pooled effect (θ = 8.69; 95% CI, 6.08–11.31; *p* < 0.001) remained stable, indicating that no single study disproportionately influenced the meta‐analytic results (Supporting Information S1: Figure 1).

### 3.5 Meta‐Regression Analysis

Meta‐regression was performed as an exploratory analysis because only six studies contributed to the quantitative synthesis. Model type, validation strategy, and event rate were not significantly associated with heterogeneity, but these findings should be interpreted cautiously given the limited number of studies and heterogeneity in AI tasks, including detection, classification, and segmentation. Log‐transformed sample size appeared to be associated with between‐study variability, suggesting that smaller data sets may have contributed to less stable and potentially more optimistic performance estimates. Residual heterogeneity remained after adjustment, indicating that unmeasured factors such as data set overlap, image acquisition protocols, annotation standards, internal validation methods, and population differences may also have influenced the pooled estimates. Therefore, the meta‐regression findings should be considered hypothesis‐generating rather than confirmatory (Supporting Information S1: Table 2).

### 3.6 Publication Bias

**Figure 4:**
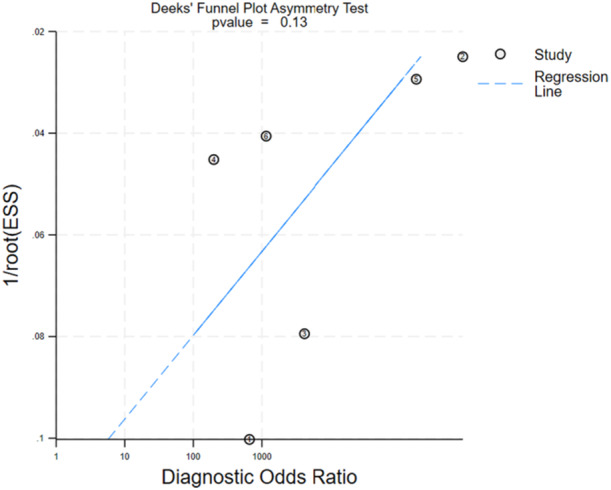
Funnel plot for publication bias.

Publication bias was assessed using Deeks' funnel plot asymmetry test (*p* = 0.13), suggesting no statistically significant small‐study effects; however, the low number of studies limits the power of this test (Figure 4).

### 3.7 Quality Assessment

Detailed PROBAST‐AI domains are available in Supporting Information S1: Table 3. For model development, the participants' domain was judged as low risk in three studies, moderate risk in four, and high risk in three. Studies rated as moderate or high frequently relied on retrospective, single‐center cohorts with unclear recruitment strategies, non‐consecutive sampling, or limited reporting of inclusion and exclusion criteria, raising concerns about selection bias and representativeness. The predictors and outcomes domains were predominantly rated as low risk (10 studies each), reflecting clearly defined imaging‐derived predictors available at the time of model application and the use of appropriate reference standards with consistent outcome definitions. In contrast, the analyses domain showed methodological limitations, with six studies rated as moderate risk due to insufficient reporting of internal validation procedures, limited assessment of calibration, inadequate handling of class imbalance, or incomplete description of missing data management. Consequently, the overall risk of bias for model development was rated as low in four studies and moderate in six, primarily driven by analytical shortcomings and participant selection issues.

For model evaluation, the risk of bias related to participants was low in one study, moderate in five, and high in four. High‐risk judgments were mainly attributable to small or highly selected validation cohorts, lack of clarity regarding patient recruitment, or substantial differences between development and evaluation populations. The predictors and outcomes domains again demonstrated low risk across most studies (10 studies each), indicating appropriate feature availability and valid outcome ascertainment. Similar to the development phase, the analyses domain was frequently rated as moderate risk (six studies), owing to incomplete reporting of performance metrics, lack of CI, absence of prespecified thresholds, or limited evaluation of calibration. As a result, the overall risk of bias for model evaluation was considered low in one study, moderate in six studies, and high in three studies.

Regarding applicability, most studies were broadly aligned with the review question. The participants' domain showed low concern in nine studies and moderate concern in one, primarily due to restricted patient populations or single‐center settings that may limit generalizability. Predictors and outcomes demonstrated low applicability concerns in all studies, as the imaging modalities and target conditions were consistent with the intended clinical use. The analyses domain raised moderate applicability concerns in six studies, largely related to limited transparency in model implementation, insufficient reporting to enable replication, or lack of information relevant to clinical deployment. Overall applicability was judged as low concern in three studies and moderate concern in seven.

**Figure 5:**
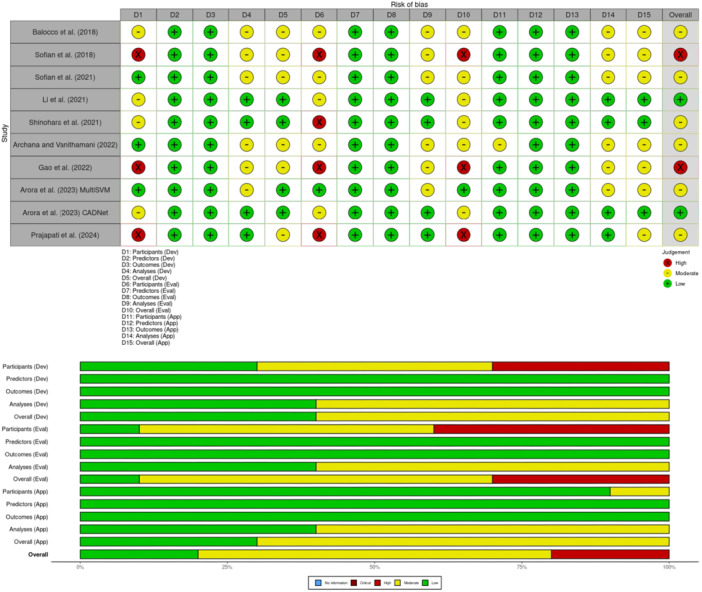
Traffic light for quality assessment.

Across all domains, the overall PROBAST‐AI judgment indicated low risk of bias in two studies, moderate risk in six studies, and high risk in two studies. The primary sources of bias were related to participant selection and analytical methods rather than predictor definition or outcome assessment (Figure 5).

### 3.8 GRADE Assessment

Across the included studies, concerns were identified regarding risk of bias, as most investigations used retrospective designs with limited reporting on patient selection procedures and model validation strategies; although appropriate reference standards were generally applied, potential selection bias and unclear handling of missing data warranted downgrading. Moderate inconsistency was also observed, reflected by a generalized *I* ^2^ of 46.12%, with heterogeneity affecting both sensitivity (*I* ^2^ = 55.39%) and specificity (*I* ^2^ = 45.35%); despite this variability, pooled estimates remained directionally consistent across studies. No serious concerns were identified for indirectness, as the populations, index tests, reference standards, and target conditions were directly aligned with the review question. Imprecision was considered acceptable, given the narrow CI around pooled sensitivity and specificity estimates, although the limited number of studies restricted certainty regarding subgroup analyses and broader generalizability. Overall model performance was good, with high pooled diagnostic accuracy and stable results on leave‐one‐out sensitivity analyses, supporting confidence in the observed effect estimates. Taken together, considering limitations related to risk of bias and heterogeneity, the overall certainty of evidence was rated as moderate (Supporting Information S1: Table 4).

## 4 Discussion

This systematic review and meta‐analysis evaluated AI‐based methods for the detection of coronary plaques using IVUS imaging and identified good diagnostic performance across the included studies. However, these estimates should not be interpreted as definitive clinical accuracy. Most included studies used retrospective data sets, small patient samples, enriched image‐level data sets, internal validation, or repeated use of public data sets. These design features may increase the risk of overfitting, data leakage, and optimistic performance estimation. Therefore, the pooled estimates primarily reflect performance under controlled research conditions. Although leave‐one‐out sensitivity analyses suggested that the pooled estimates were not driven by any single study, the restricted evidence base and methodological variability introduce uncertainty regarding the robustness and generalizability of these findings to broader clinical practice.

Recent advances in coronary imaging increasingly emphasize biological plaque characterization rather than luminal stenosis alone, including CT‐derived assessment of high‐risk plaque morphology and pericoronary adipose tissue inflammation. Although these approaches differ from IVUS, they support the broader clinical rationale for automated, reproducible plaque phenotyping across coronary imaging modalities [22].

Deep learning‐based architectures accounted for the majority of included models and generally outperformed conventional machine learning approaches. Residual network‐based classifiers enhanced with attention mechanisms achieved strong plaque‐type recognition, with classification accuracy reaching 87.1% across three plaque phenotypes using 720 IVUS images, exceeding the performance of texture‐based GLCM features combined with support vector machines by more than 10 percentage points [20]. These findings underscore the advantage of end‐to‐end feature learning over handcrafted descriptors in IVUS data.

Encoder–decoder architectures, particularly U‐Net and its variants, were predominantly applied to segmentation tasks and demonstrated high spatial agreement with expert annotations. Cascaded and attention‐augmented U‐Net models achieved mean IoU values ranging from approximately 0.66 to 0.79 and Dice coefficients exceeding 0.87 for calcified plaque segmentation in data sets comprising between 1097 and 3738 IVUS frames [16, 18, 21]. Notably, segmentation accuracy was highest for lumen boundaries, with IoU values approaching 0.86, while performance declined in regions affected by stent artifacts or severe acoustic shadowing, highlighting modality‐specific challenges [17].

Several studies employed transfer learning strategies to address limited sample sizes, combining pretrained convolutional backbones with classical classifiers. Using deep features extracted from AlexNet and optimized with multi‐class support vector machines, near‐perfect classification accuracy of 99.8% was reported for calcification severity grading, albeit in a small data set derived from IVUS pullbacks [13]. Similarly, deep feature learning with ResNet and Inception architectures achieved accuracy values approaching 100% under cross‐validation, although these results were derived from data sets comprising fewer than 2200 images, raising concerns regarding generalizability [15].

More complex ensemble and hybrid frameworks incorporating bio‐inspired optimization techniques further reported high diagnostic accuracy. The AAPC‐HALODL model achieved classification accuracy exceeding 98% by integrating region‐based convolutional networks, lightweight feature extractors, and Ant Lion Optimizer‐driven hyperparameter tuning [8]. While these results are promising, the increased architectural complexity and computational requirements may limit real‐time clinical deployment.

## 5 Clinical Implications

AI‐assisted IVUS analysis may support more standardized and efficient assessment of coronary plaque and calcification, particularly in complex PCI, where calcium burden, plaque morphology, and acoustic shadowing influence procedural planning. Potential clinical applications include automated frame selection, calcification detection, plaque‐type classification, and segmentation of the lumen, vessel wall, and calcium‐related shadow borders. Integration with complementary intracoronary imaging modalities may further improve plaque characterization, as cross‐validated deep learning approaches have shown high agreement between OCT and IVUS references [23].

This potential is clinically relevant because severe coronary calcification can impair stent delivery, expansion, and apposition, increasing the risk of procedural complications, stent underexpansion, thrombosis, and restenosis [24]. AI‐based IVUS tools may also help quantify plaque burden and morphology more consistently, supporting lesion‐level risk assessment and procedural planning in patients with complex coronary disease [25, 26]. However, current evidence remains insufficient for direct clinical deployment. Most studies were retrospective, relied on limited patient samples, used internal validation, and did not evaluate performance across different centers, IVUS systems, catheter frequencies, operators, lesion subsets, or real‐time catheterization laboratory workflows.

For clinical translation, future AI‐IVUS studies should follow TRIPOD + AI reporting standards, including clear specification of the intended clinical use, target population, data sources, preprocessing pipeline, annotation process, model architecture, data‐splitting strategy, handling of class imbalance, calibration, uncertainty estimates, and external validation [27]. Particular attention should be given to patient‐ or pullback‐level data separation to reduce the risk of data leakage and overly optimistic performance estimates. Before integration into catheterization laboratory workflows, AI‐IVUS models require prospective multicenter validation, assessment of processing time, comparison against expert readers, and evidence that model outputs improve clinical decisions or procedural outcomes.

### 5.1 Study Limitations

This review has several limitations. First, only six studies provided sufficient data for quantitative synthesis, limiting the precision of pooled estimates and the reliability of subgroup or meta‐regression analyses. Second, the included studies evaluated heterogeneous AI tasks, including calcified plaque detection, plaque‐type classification, and segmentation; therefore, pooled diagnostic estimates should not be interpreted as a single measure of performance for all IVUS plaque‐analysis applications. Third, partial data set overlap was present because several studies used the MICCAI 2011 Data set B, reducing study independence and potentially inflating pooled accuracy. Fourth, most studies used retrospective designs, small patient cohorts, enriched image‐level data sets, and internal validation strategies, which may increase the risk of overfitting, data leakage, and selection bias. Fifth, external validation was uncommon, and few studies assessed calibration, clinical decision thresholds, processing time, model reproducibility, or performance across IVUS vendors and acquisition protocols. These limitations mean that the high pooled sensitivity, specificity, and AUROC should be interpreted as preliminary evidence of technical feasibility rather than proof of clinical effectiveness.

### 5.2 Future Perspectives

Looking ahead, the field of AI‐assisted IVUS for coronary plaque detection stands at a pivotal juncture, with opportunities to address current gaps and realize broader clinical impact. Priority should be given to large‐scale, prospective, multicenter studies incorporating external validation across diverse populations and real‐world acquisitions to mitigate bias and enhance generalizability [28, 29]. Integration of multimodal data, like fusing IVUS with OCT, near‐infrared spectroscopy (NIRS), or noninvasive coronary CT angiography, could yield hybrid AI models for comprehensive plaque characterization, overcoming individual modality limitations. For clinical translation, future AI‐IVUS studies should follow TRIPOD + AI reporting standards, including clear specification of the intended clinical use, target population, data sources, preprocessing pipeline, annotation process, model architecture, data‐splitting strategy, handling of class imbalance, calibration, uncertainty estimates, and external validation [27].

## 6 Conclusion

AI‐based IVUS analysis demonstrated high reported diagnostic performance for coronary plaque and calcification assessment in the available literature. However, the evidence base is small and methodologically heterogeneous, with frequent reliance on retrospective data sets, internal validation, limited patient‐level sample sizes, and partial reuse of public data sets. The pooled estimates, therefore, represent promising technical performance under selected research conditions rather than definitive evidence of real‐world clinical accuracy. Future studies should prioritize prospective multicenter external validation, patient‐ and pullback‐level data separation, transparent TRIPOD + AI reporting, calibration assessment, and evaluation of clinical workflow integration before AI‐assisted IVUS plaque analysis can be recommended for routine practice.

## Author Contributions

P.E. was the primary contributor in the design, implementation, and writing of the manuscript. M.R., H.S., and M.K. independently assessed articles and extracted data. All authors read and approved the final manuscript.

## Funding

The authors have nothing to report.

## Ethics Statement

The authors have nothing to report.

## Consent

The authors have nothing to report.

## Conflicts of Interest

The authors declare no conflicts of interest.

## Supporting information


Supporting File

## Data Availability

All data are included in the manuscript and supplementary files.
