# Supplementary material for: Artificial Intelligence‐Based Analysis of Coronary Atherosclerotic Plaque on Intravascular Ultrasound: A Systematic Review and Meta‐Analysis
Source: Clin Cardiol. 2026 Jul 21;49(7):e70423. doi: 10.1002/clc.70423 (PMC13386478; doi:10.1002/clc.70423)
Supplement: Supplementary file 1 — Supporting File [file CLC-49-e70423-s001.docx]

| **Supplementary Table 1. Search syntax for different databases.** | | |
| --- | --- | --- |
| Database | Syntax | Results |
| Pubmed | ("Machine learning" OR "Machine Learning"[Mesh] OR "artificial intelligence" OR "deep learning" OR "prediction model*" ) AND ("Interventional Ultrasonography" OR "ivus" OR "Interventional Ultrasound" OR "Intravascular Ultrasonography" OR "Ultrasonography, Interventional"[Mesh]) | 476 |
| WEB OF SCIENCE | TS=("machine learning" OR "artificial intelligence" OR "deep learning" OR "prediction model*") AND TS=("interventional ultrasonography" OR ivus OR "interventional ultrasound" OR "intravascular ultrasonography") | 351 |
| Scopus | (TITLE-ABS-KEY("machine learning" OR "artificial intelligence" OR "deep learning" OR "prediction model*")) AND (TITLE-ABS-KEY("interventional ultrasonography" OR "ivus" OR "interventional ultrasound" OR "intravascular ultrasonography")) | 436 |
| EMBASE | ('machine learning'/de OR 'machine learning' OR 'artificial intelligence'/de OR 'artificial intelligence' OR 'deep learning'/de OR 'deep learning' OR 'prediction model*':ti,ab,kw) AND ('interventional ultrasonography'/de OR 'interventional ultrasonography' OR 'interventional ultrasound' OR 'intravascular ultrasonography'/de OR 'intravascular ultrasonography' OR ivus:ti,ab,kw) | 435 |
| Ebsco | (MH "Machine Learning" OR "machine learning" OR "artificial intelligence" OR "deep learning" OR "prediction model*") AND (MH "Ultrasonography, Interventional" OR "interventional ultrasonography" OR "interventional ultrasound" OR ivus OR "intravascular ultrasonography") | 18 |
| Total |  | 1716 |

| **Supplementary Table 2. Meta-regression results for sources of heterogeneity** | | | |
| --- | --- | --- | --- |
| **Covariate** | **Likelihood ratio χ²** | **P value** | **Residual I² (%)** |
| Model type | 1.64 | 0.44 | 0 |
| Validation type | 2.54 | 0.28 | 21 |
| Log-transformed sample size | 6.36 | 0.04 | 69 |
| Event rate | 1.07 | 0.59 | 0 |

| 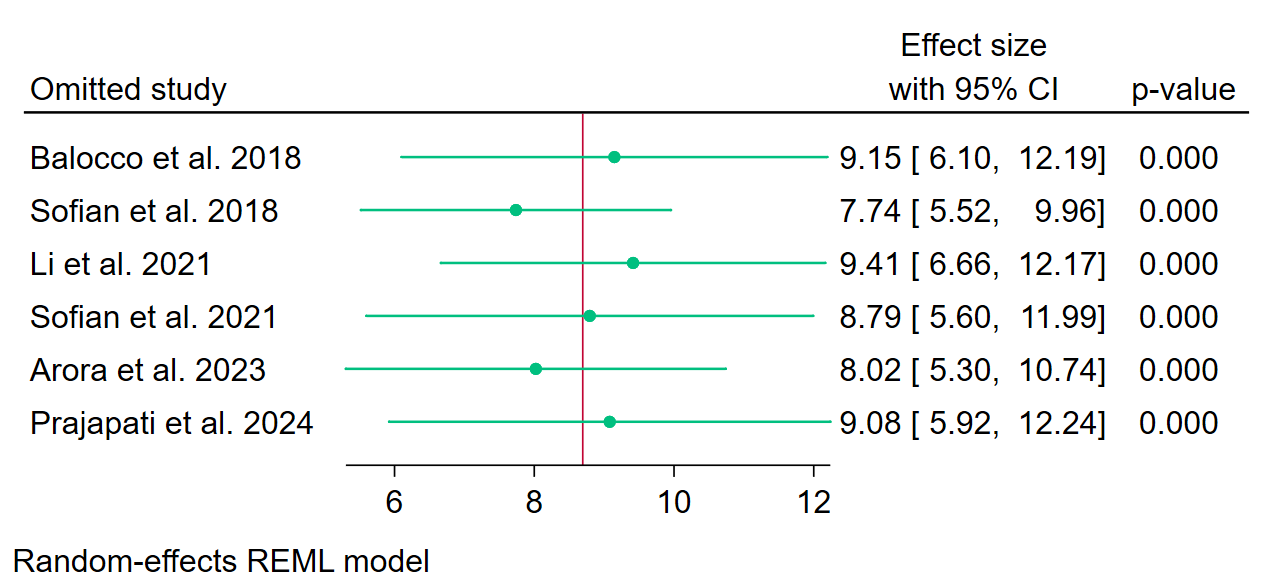 |
| --- |
| **Supplementary figure 1. leave-one-out sensitivity analysis.** |

| **Supplementary Table 3. Quality assessment checklist based on the PROBAST+AI tool** |
| --- |
| **Development Domains**  **Participants and data sources — Development**  1. Were participants, IVUS pullbacks, or image frames in the development dataset representative of the intended target population, including patients undergoing IVUS imaging for suspected or established coronary artery disease, atherosclerosis, or coronary calcification?  **Predictors — Development** 2. Were model predictors, including IVUS image features, extracted frames, pullback-level data, preprocessing steps, and augmentation procedures, clearly defined, consistently applied, and available at the time the AI model would be used?  **Outcome — Development** 3. Was the target outcome clearly defined, such as calcified plaque presence, plaque type, plaque burden, lumen/vessel wall segmentation, or calcification/shadow-border segmentation, using an appropriate reference standard such as expert annotation or consensus labeling?  **Analysis — Development** 4. Were AI model development methods appropriate and transparent, including algorithm selection, data splitting, hyperparameter tuning, cross-validation, handling of class imbalance, missing data management, and prevention of data leakage between training and validation datasets?  **Overall risk of bias — Development** 5. Was the overall model development process likely to produce an unbiased and reproducible model for IVUS-based coronary plaque or calcification assessment? |
| **Evaluation Domains**  **Participants and data sources — Evaluation** 6. Were participants, IVUS pullbacks, or image frames in the evaluation dataset representative of the target clinical population, and were they sufficiently independent from the development dataset?  **Predictors — Evaluation** 7. Were predictors in the evaluation dataset obtained and processed in the same way as in the development dataset, without using information from the reference standard or outcome labels?  **Outcome — Evaluation** 8. Was the outcome in the evaluation dataset defined and assessed using the same plaque or calcification reference standard as in model development, preferably by blinded expert readers or consensus annotation?  **Analysis — Evaluation** 9. Were evaluation methods appropriate, including patient-level or pullback-level separation where possible, reporting of sensitivity, specificity, accuracy, AUC, Dice coefficient, IoU, or other relevant metrics with uncertainty measures?  **Overall risk of bias — Evaluation** 10. Was the evaluation process sufficiently independent, transparent, and methodologically robust to provide reliable estimates of model performance for IVUS-based coronary plaque analysis? |
| **Applicability Domains**  **Participants and data sources — Applicability** 11. Did the study population, clinical setting, IVUS acquisition protocol, catheter type or frequency, and disease spectrum match the intended use of AI-assisted IVUS plaque assessment in patients with coronary artery disease?  **Predictors — Applicability** 12. Were the required predictors, including IVUS images, pullbacks, preprocessing inputs, and annotation requirements, feasible and available in routine catheterization laboratory or research settings?  **Outcome — Applicability** 13. Did the target outcome correspond to clinically meaningful IVUS findings, such as calcified plaque detection, plaque-type characterization, lumen or vessel wall segmentation, or calcium-related acoustic shadow assessment?  **Analysis — Applicability** 14. Did the reported analysis support clinical translation, including reproducibility, external validation, robustness across datasets, handling of artifacts or stents, processing time, and potential integration into IVUS interpretation workflows?  **Overall applicability** 15. Were the study design, population, predictors, outcomes, and analysis methods sufficiently aligned with the review question and the intended clinical use of AI-based IVUS plaque or calcification assessment? |

| **Supplementary table 4. Summary of GRADE Assessment.** | | |
| --- | --- | --- |
| **GRADE Domain** | **Judgment** | **Rationale** |
| Risk of bias | Serious concerns | Predominantly retrospective designs and incomplete reporting of selection and validation processes |
| Inconsistency (heterogeneity) | Serious concerns | Moderate heterogeneity across studies (generalized I² = 46.12%) |
| Indirectness | No serious concerns | Direct applicability of populations, index tests, and outcomes |
| Imprecision | No serious concerns | Narrow confidence intervals, but limited number of studies |
| Publication bias | No Serious concerns | No Evidence of small-study effects |
| Overall model performance | Consistent | Stable pooled estimates and sensitivity analyses |
| **Overall certainty of evidence** | **Moderate** | Downgraded due to bias, heterogeneity, and publication bias |
